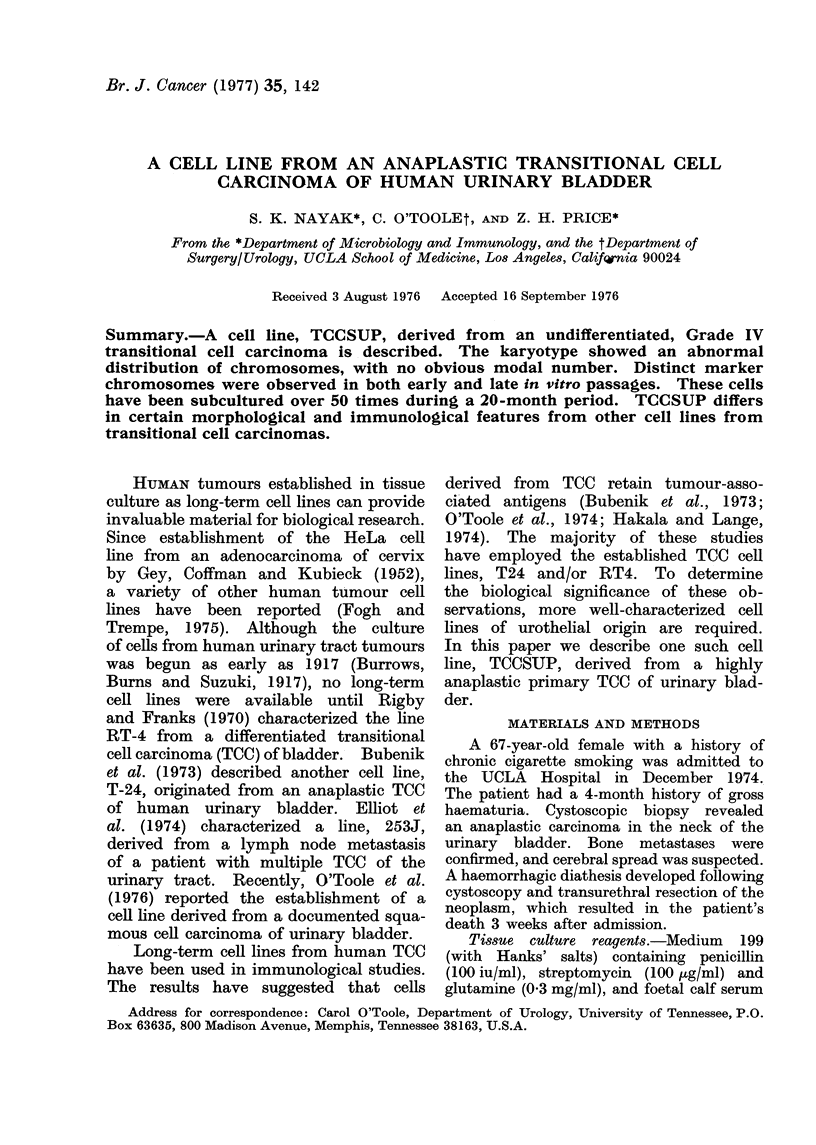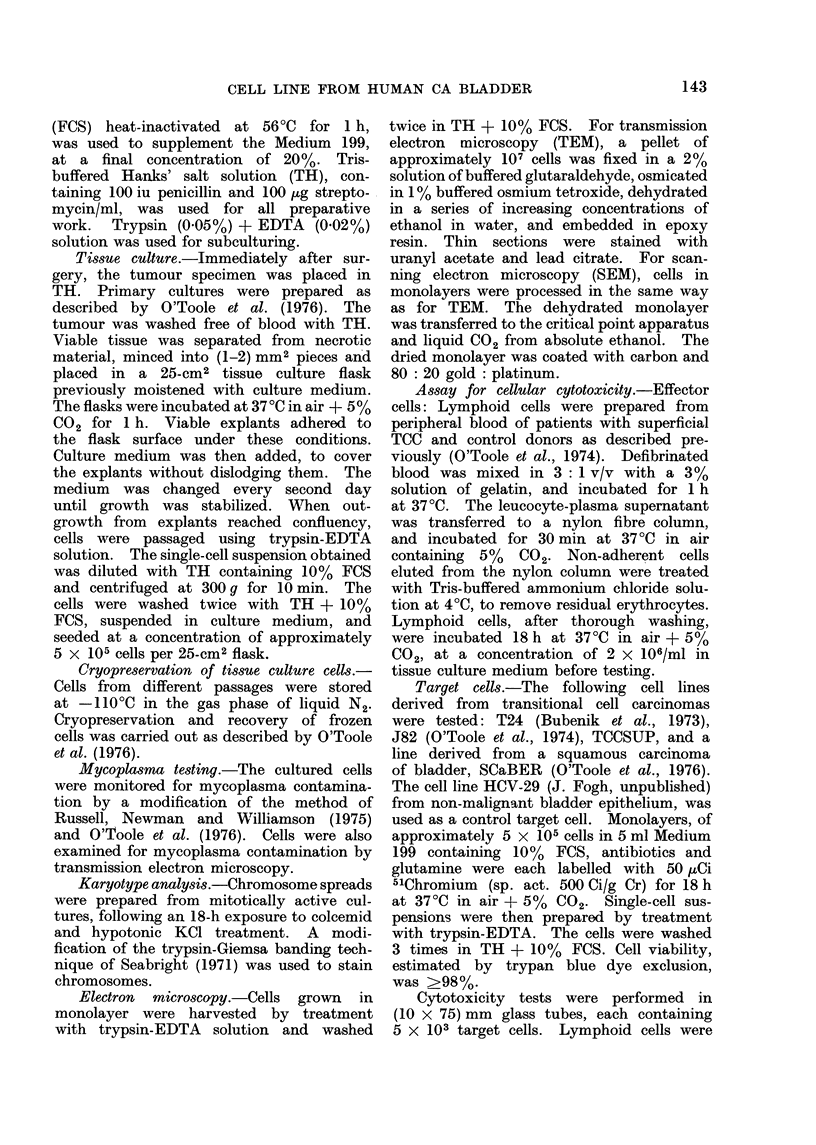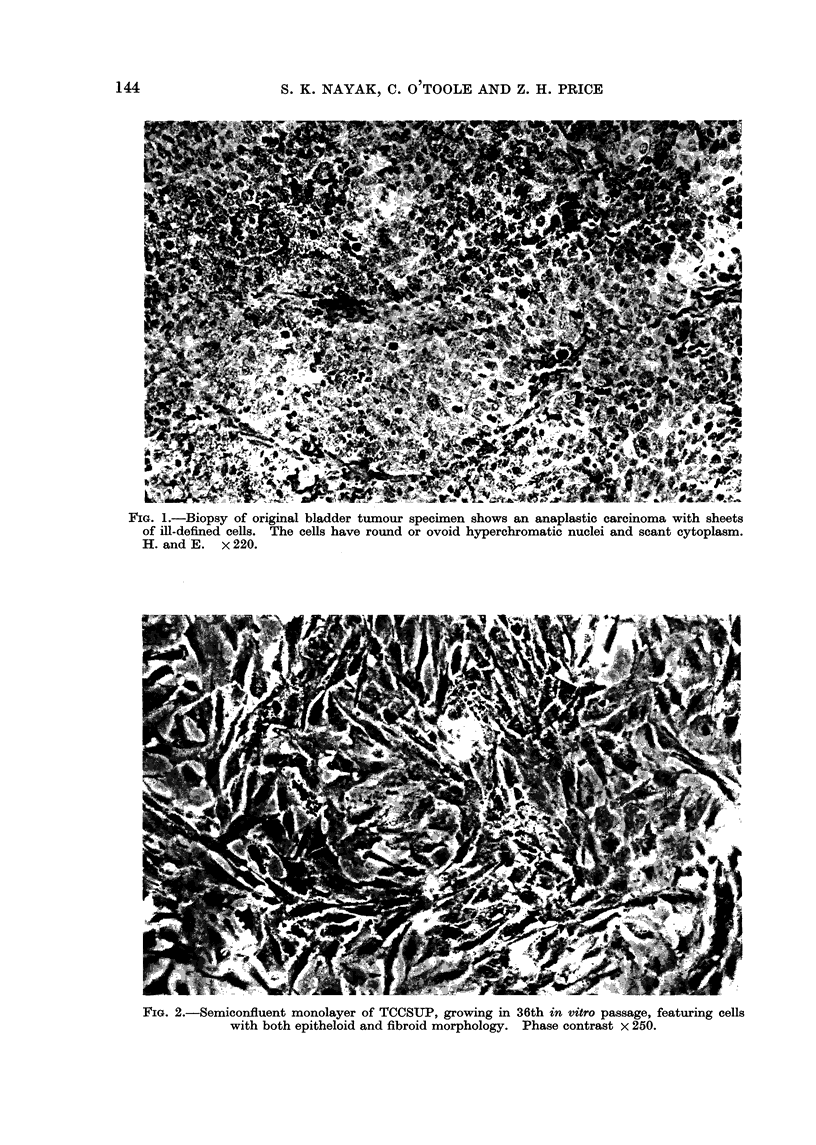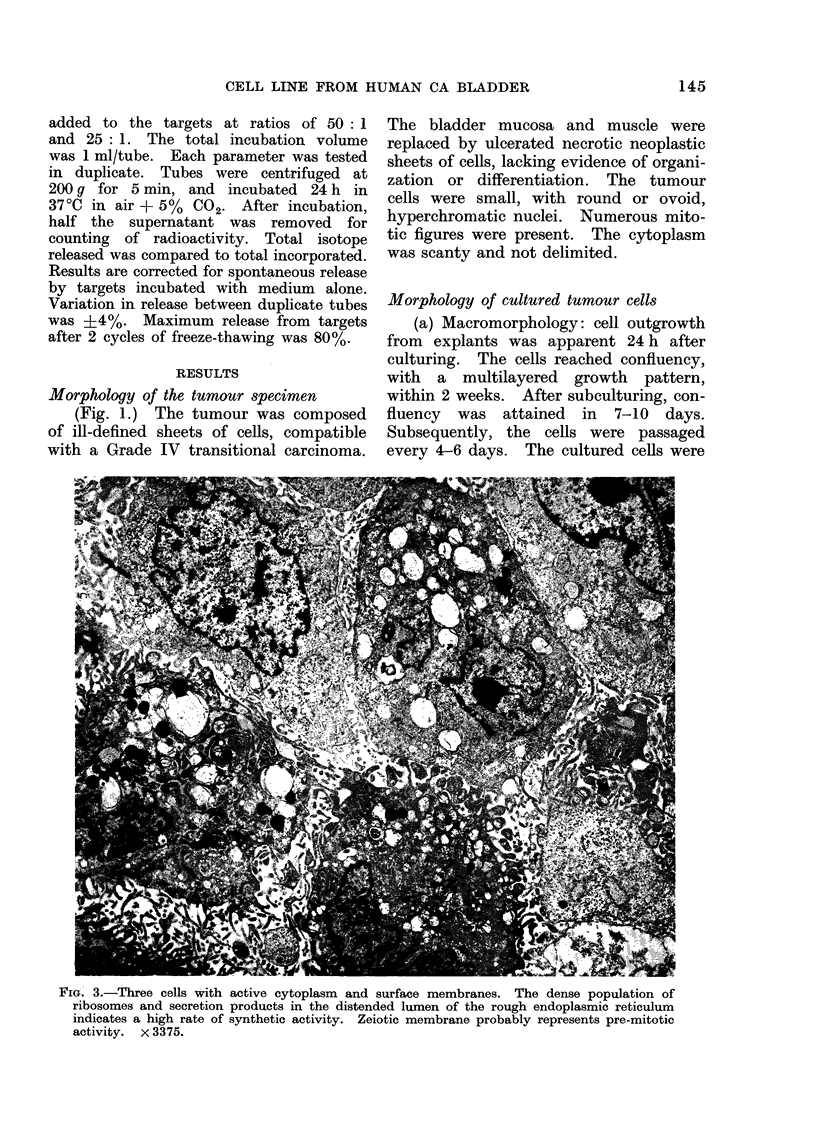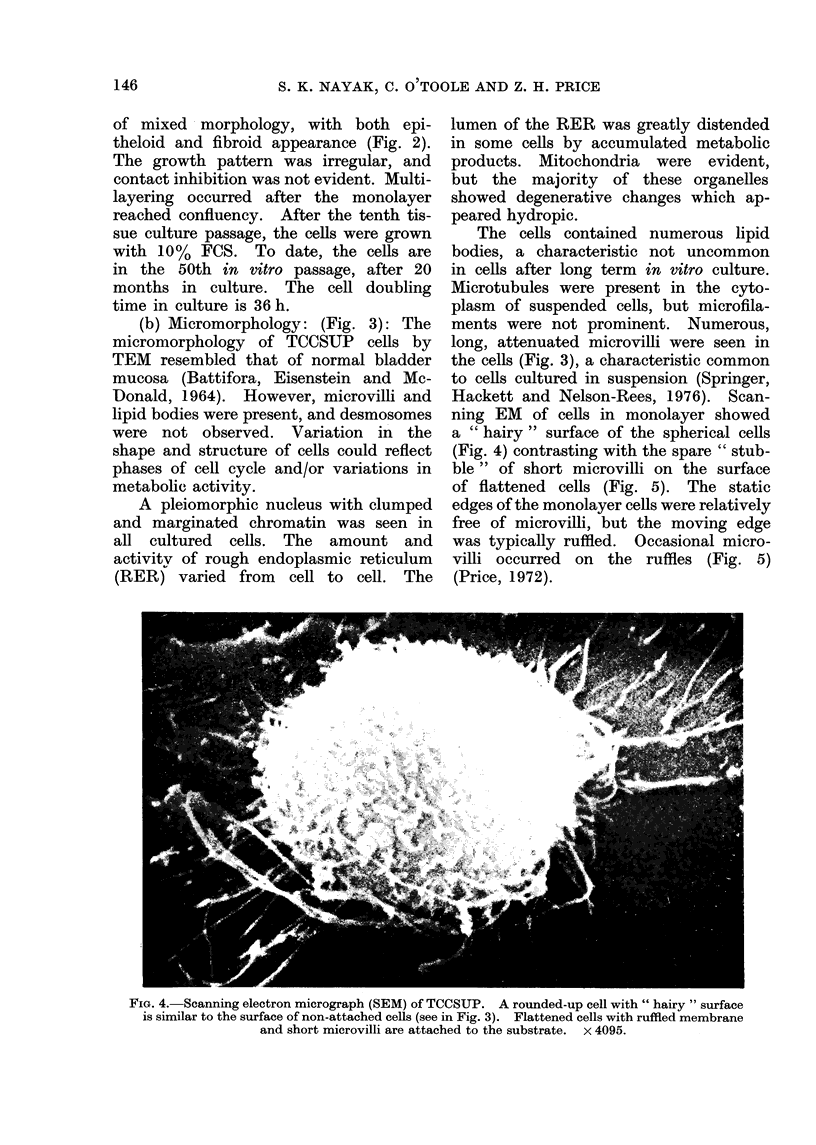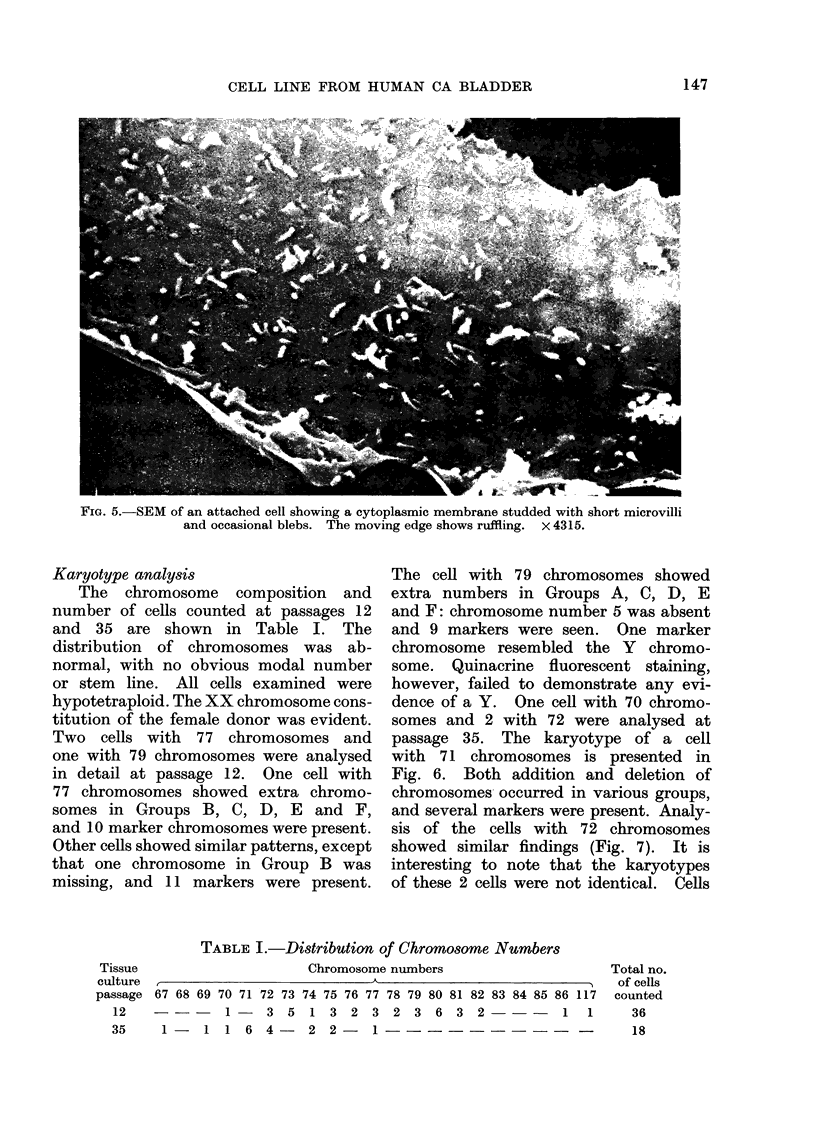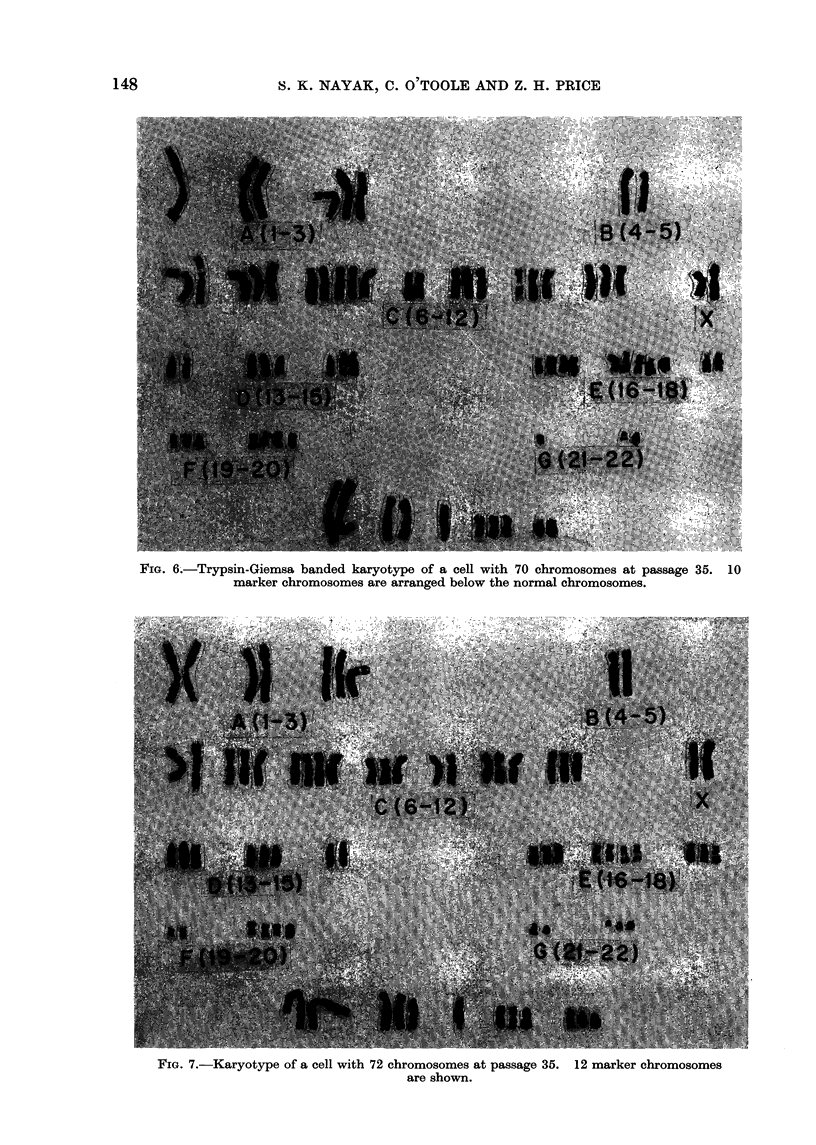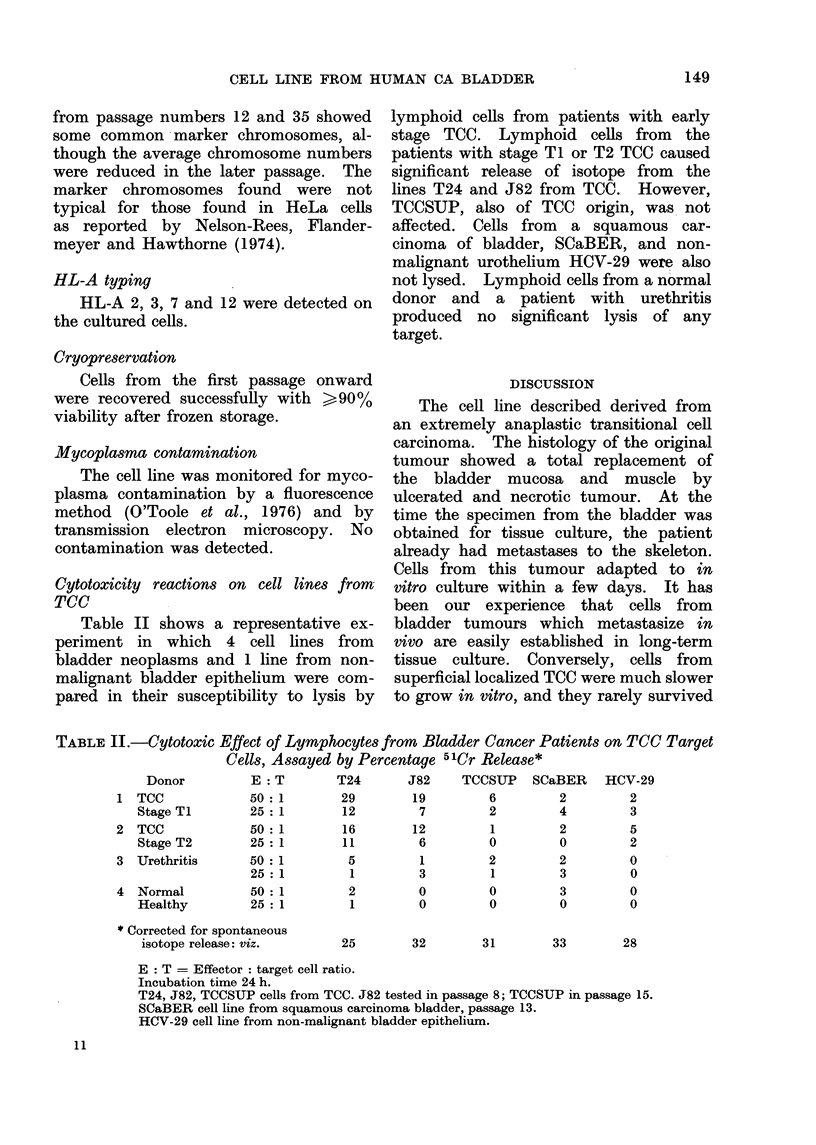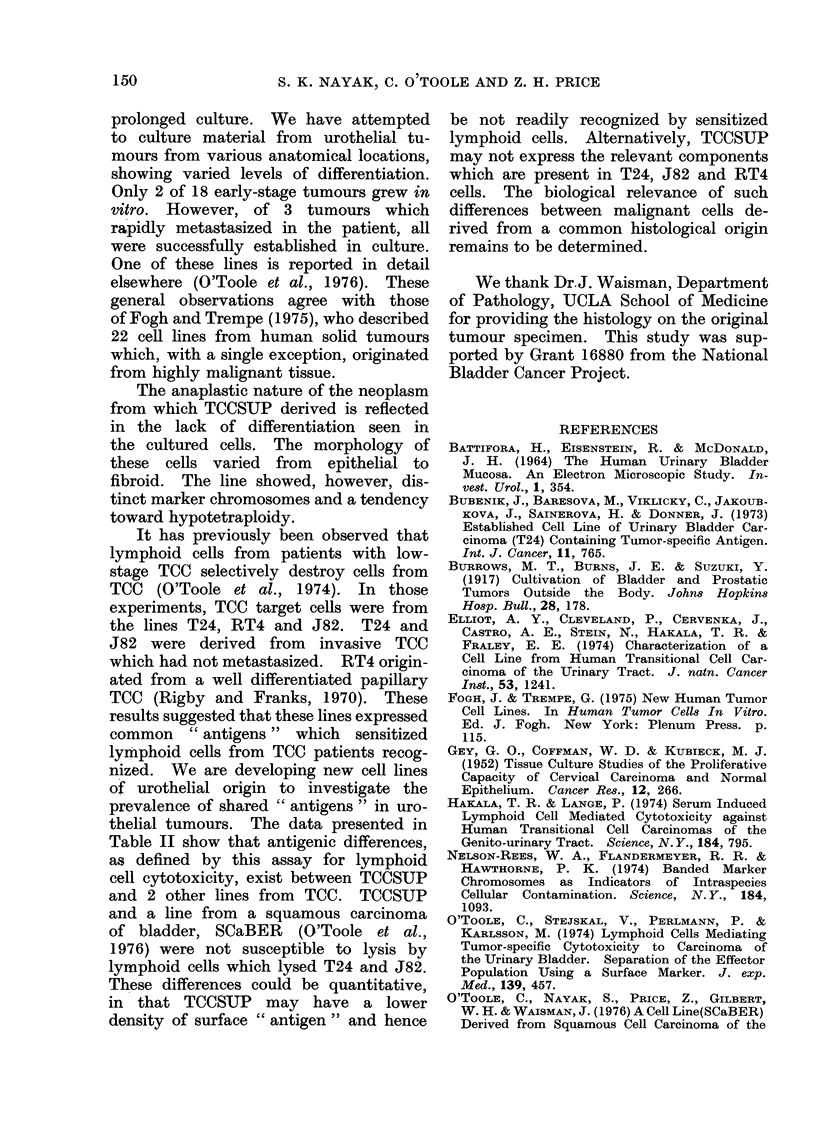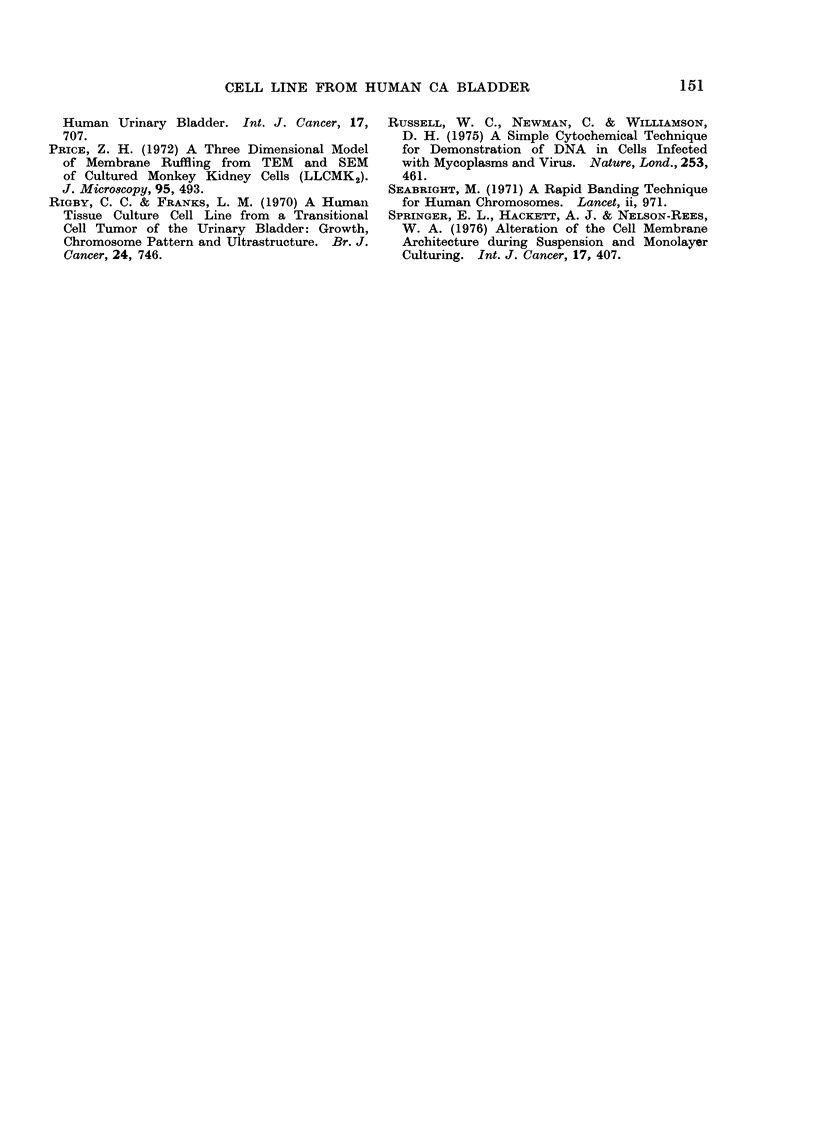# A cell line from an anaplastic transitional cell carcinoma of human urinary bladder.

**DOI:** 10.1038/bjc.1977.21

**Published:** 1977-02

**Authors:** S. K. Nayak, C. O'Toole, Z. H. Price

## Abstract

**Images:**


*Br. J. Cancer* (1977) **35**, 142

### A CELL LINE FROM AN ANAPLASTIC TRANSITIONAL CELL CARCINOMA OF HUMAN URINARY BLADDER

S. K. NAYAK*, C. O'TOOLE†, AND Z. H. PRICE*

*From the *Department of Microbiology and Immunology, and the †Department of Surgery/Urology, UCLA School of Medicine, Los Angeles, California* 90024

Received 3 August 1976 Accepted 16 September 1976

**Summary.—A cell line, TCCSUP, derived from an undifferentiated, Grade IV transitional cell carcinoma is described. The karyotype showed an abnormal distribution of chromosomes, with no obvious modal number. Distinct marker chromosomes were observed in both early and late *in vitro* passages. These cells have been subcultured over 50 times during a 20-month period. TCCSUP differs in certain morphological and immunological features from other cell lines from transitional cell carcinomas.**

HUMAN tumours established in tissue culture as long-term cell lines can provide invaluable material for biological research. Since establishment of the HeLa cell line from an adenocarcinoma of cervix by Gey, Coffman and Kubieck (1952), a variety of other human tumour cell lines have been reported (Fogh and Trempe, 1975). Although the culture of cells from human urinary tract tumours was begun as early as 1917 (Burrows, Burns and Suzuki, 1917), no long-term cell lines were available until Rigby and Franks (1970) characterized the line RT-4 from a differentiated transitional cell carcinoma (TCC) of bladder. Bubenik *et al.* (1973) described another cell line, T-24, originated from an anaplastic TCC of human urinary bladder. Elliot *et al.* (1974) characterized a line, 253J, derived from a lymph node metastasis of a patient with multiple TCC of the urinary tract. Recently, O'Toole *et al.* (1976) reported the establishment of a cell line derived from a documented squamous cell carcinoma of urinary bladder.

Long-term cell lines from human TCC have been used in immunological studies. The results have suggested that cells derived from TCC retain tumour-associated antigens (Bubenik *et al.*, 1973; O'Toole *et al.*, 1974; Hakala and Lange, 1974). The majority of these studies have employed the established TCC cell lines, T24 and/or RT4. To determine the biological significance of these observations, more well-characterized cell lines of urothelial origin are required. In this paper we describe one such cell line, TCCSUP, derived from a highly anaplastic primary TCC of urinary bladder.

### MATERIALS AND METHODS

A 67-year-old female with a history of chronic cigarette smoking was admitted to the UCLA Hospital in December 1974. The patient had a 4-month history of gross haematuria. Cystoscopic biopsy revealed an anaplastic carcinoma in the neck of the urinary bladder. Bone metastases were confirmed, and cerebral spread was suspected. A haemorrhagic diathesis developed following cystoscopy and transurethral resection of the neoplasm, which resulted in the patient's death 3 weeks after admission.

*Tissue culture reagents.*—Medium 199 (with Hanks' salts) containing penicillin (100 iu/ml), streptomycin (100 $\mu$g/ml) and glutamine (0·3 mg/ml), and foetal calf serum

Address for correspondence: Carol O'Toole, Department of Urology, University of Tennessee, P.O. Box 63635, 800 Madison Avenue, Memphis, Tennessee 38163, U.S.A.

(FCS) heat-inactivated at 56°C for 1 h, was used to supplement the Medium 199, at a final concentration of 20%. Tris-buffered Hanks' salt solution (TH), containing 100 iu penicillin and 100 μg streptomycin/ml, was used for all preparative work. Trypsin (0·05%) + EDTA (0·02%) solution was used for subculturing.

*Tissue culture.*—Immediately after surgery, the tumour specimen was placed in TH. Primary cultures were prepared as described by O'Toole *et al.* (1976). The tumour was washed free of blood with TH. Viable tissue was separated from necrotic material, minced into (1–2) $mm^2$ pieces and placed in a 25-$cm^2$ tissue culture flask previously moistened with culture medium. The flasks were incubated at 37°C in air + 5% $CO_2$ for 1 h. Viable explants adhered to the flask surface under these conditions. Culture medium was then added, to cover the explants without dislodging them. The medium was changed every second day until growth was stabilized. When outgrowth from explants reached confluency, cells were passaged using trypsin-EDTA solution. The single-cell suspension obtained was diluted with TH containing 10% FCS and centrifuged at 300 *g* for 10 min. The cells were washed twice with TH + 10% FCS, suspended in culture medium, and seeded at a concentration of approximately $5 \times 10^5$ cells per 25-$cm^2$ flask.

*Cryopreservation of tissue culture cells.*—Cells from different passages were stored at −110°C in the gas phase of liquid $N_2$. Cryopreservation and recovery of frozen cells was carried out as described by O'Toole *et al.* (1976).

*Mycoplasma testing.*—The cultured cells were monitored for mycoplasma contamination by a modification of the method of Russell, Newman and Williamson (1975) and O'Toole *et al.* (1976). Cells were also examined for mycoplasma contamination by transmission electron microscopy.

*Karyotype analysis.*—Chromosome spreads were prepared from mitotically active cultures, following an 18-h exposure to colcemid and hypotonic KCl treatment. A modification of the trypsin-Giemsa banding technique of Seabright (1971) was used to stain chromosomes.

*Electron microscopy.*—Cells grown in monolayer were harvested by treatment with trypsin-EDTA solution and washed twice in TH + 10% FCS. For transmission electron microscopy (TEM), a pellet of approximately $10^7$ cells was fixed in a 2% solution of buffered glutaraldehyde, osmicated in 1% buffered osmium tetroxide, dehydrated in a series of increasing concentrations of ethanol in water, and embedded in epoxy resin. Thin sections were stained with uranyl acetate and lead citrate. For scanning electron microscopy (SEM), cells in monolayers were processed in the same way as for TEM. The dehydrated monolayer was transferred to the critical point apparatus and liquid $CO_2$ from absolute ethanol. The dried monolayer was coated with carbon and 80 : 20 gold : platinum.

*Assay for cellular cytotoxicity.*—Effector cells: Lymphoid cells were prepared from peripheral blood of patients with superficial TCC and control donors as described previously (O'Toole *et al.*, 1974). Defibrinated blood was mixed in 3 : 1 v/v with a 3% solution of gelatin, and incubated for 1 h at 37°C. The leucocyte-plasma supernatant was transferred to a nylon fibre column, and incubated for 30 min at 37°C in air containing 5% $CO_2$. Non-adherent cells eluted from the nylon column were treated with Tris-buffered ammonium chloride solution at 4°C, to remove residual erythrocytes. Lymphoid cells, after thorough washing, were incubated 18 h at 37°C in air + 5% $CO_2$, at a concentration of $2 \times 10^6$/ml in tissue culture medium before testing.

*Target cells.*—The following cell lines derived from transitional cell carcinomas were tested: T24 (Bubenik *et al.*, 1973), J82 (O'Toole *et al.*, 1974), TCCSUP, and a line derived from a squamous carcinoma of bladder, SCaBER (O'Toole *et al.*, 1976). The cell line HCV-29 (J. Fogh, unpublished) from non-malignant bladder epithelium, was used as a control target cell. Monolayers, of approximately $5 \times 10^5$ cells in 5 ml Medium 199 containing 10% FCS, antibiotics and glutamine were each labelled with 50 μCi $^{51}$Chromium (sp. act. 500 Ci/g Cr) for 18 h at 37°C in air + 5% $CO_2$. Single-cell suspensions were then prepared by treatment with trypsin-EDTA. The cells were washed 3 times in TH + 10% FCS. Cell viability, estimated by trypan blue dye exclusion, was $\geq$98%.

Cytotoxicity tests were performed in (10 × 75) mm glass tubes, each containing $5 \times 10^3$ target cells. Lymphoid cells were

FIG. 1.—Biopsy of original bladder tumour specimen shows an anaplastic carcinoma with sheets of ill-defined cells. The cells have round or ovoid hyperchromatic nuclei and scant cytoplasm. H. and E. ×220.

FIG. 2.—Semiconfluent monolayer of TCCSUP, growing in 36th *in vitro* passage, featuring cells with both epitheloid and fibroid morphology. Phase contrast ×250.

added to the targets at ratios of 50 : 1 and 25 : 1. The total incubation volume was 1 ml/tube. Each parameter was tested in duplicate. Tubes were centrifuged at 200 $g$ for 5 min, and incubated 24 h in 37 °C in air + 5% $CO_2$. After incubation, half the supernatant was removed for counting of radioactivity. Total isotope released was compared to total incorporated. Results are corrected for spontaneous release by targets incubated with medium alone. Variation in release between duplicate tubes was ±4%. Maximum release from targets after 2 cycles of freeze-thawing was 80%.

### RESULTS

### *Morphology of the tumour specimen*

(Fig. 1.) The tumour was composed of ill-defined sheets of cells, compatible with a Grade IV transitional carcinoma. The bladder mucosa and muscle were replaced by ulcerated necrotic neoplastic sheets of cells, lacking evidence of organization or differentiation. The tumour cells were small, with round or ovoid, hyperchromatic nuclei. Numerous mitotic figures were present. The cytoplasm was scanty and not delimited.

### *Morphology of cultured tumour cells*

(a) Macromorphology: cell outgrowth from explants was apparent 24 h after culturing. The cells reached confluency, with a multilayered growth pattern, within 2 weeks. After subculturing, confluency was attained in 7–10 days. Subsequently, the cells were passaged every 4–6 days. The cultured cells were

FIG. 3.—Three cells with active cytoplasm and surface membranes. The dense population of ribosomes and secretion products in the distended lumen of the rough endoplasmic reticulum indicates a high rate of synthetic activity. Zeiotic membrane probably represents pre-mitotic activity. ×3375.

of mixed morphology, with both epitheloid and fibroid appearance (Fig. 2). The growth pattern was irregular, and contact inhibition was not evident. Multilayering occurred after the monolayer reached confluency. After the tenth tissue culture passage, the cells were grown with 10% FCS. To date, the cells are in the 50th *in vitro* passage, after 20 months in culture. The cell doubling time in culture is 36 h.

(b) Micromorphology: (Fig. 3): The micromorphology of TCCSUP cells by TEM resembled that of normal bladder mucosa (Battifora, Eisenstein and McDonald, 1964). However, microvilli and lipid bodies were present, and desmosomes were not observed. Variation in the shape and structure of cells could reflect phases of cell cycle and/or variations in metabolic activity.

A pleiomorphic nucleus with clumped and marginated chromatin was seen in all cultured cells. The amount and activity of rough endoplasmic reticulum (RER) varied from cell to cell. The lumen of the RER was greatly distended in some cells by accumulated metabolic products. Mitochondria were evident, but the majority of these organelles showed degenerative changes which appeared hydropic.

The cells contained numerous lipid bodies, a characteristic not uncommon in cells after long term *in vitro* culture. Microtubules were present in the cytoplasm of suspended cells, but microfilaments were not prominent. Numerous, long, attenuated microvilli were seen in the cells (Fig. 3), a characteristic common to cells cultured in suspension (Springer, Hackett and Nelson-Rees, 1976). Scanning EM of cells in monolayer showed a " hairy " surface of the spherical cells (Fig. 4) contrasting with the spare " stubble " of short microvilli on the surface of flattened cells (Fig. 5). The static edges of the monolayer cells were relatively free of microvilli, but the moving edge was typically ruffled. Occasional microvilli occurred on the ruffles (Fig. 5) (Price, 1972).

Fig. 4.—Scanning electron micrograph (SEM) of TCCSUP. A rounded-up cell with " hairy " surface is similar to the surface of non-attached cells (see in Fig. 3). Flattened cells with ruffled membrane and short microvilli are attached to the substrate. × 4095.

FIG. 5.—SEM of an attached cell showing a cytoplasmic membrane studded with short microvilli and occasional blebs. The moving edge shows ruffling. ×4315.

*Karyotype analysis*

The chromosome composition and number of cells counted at passages 12 and 35 are shown in Table I. The distribution of chromosomes was abnormal, with no obvious modal number or stem line. All cells examined were hypotetraploid. The XX chromosome constitution of the female donor was evident. Two cells with 77 chromosomes and one with 79 chromosomes were analysed in detail at passage 12. One cell with 77 chromosomes showed extra chromosomes in Groups B, C, D, E and F, and 10 marker chromosomes were present. Other cells showed similar patterns, except that one chromosome in Group B was missing, and 11 markers were present. The cell with 79 chromosomes showed extra numbers in Groups A, C, D, E and F: chromosome number 5 was absent and 9 markers were seen. One marker chromosome resembled the Y chromosome. Quinacrine fluorescent staining, however, failed to demonstrate any evidence of a Y. One cell with 70 chromosomes and 2 with 72 were analysed at passage 35. The karyotype of a cell with 71 chromosomes is presented in Fig. 6. Both addition and deletion of chromosomes occurred in various groups, and several markers were present. Analysis of the cells with 72 chromosomes showed similar findings (Fig. 7). It is interesting to note that the karyotypes of these 2 cells were not identical. Cells

TABLE I.—*Distribution of Chromosome Numbers*

| Tissue culture passage | Chromosome numbers | | | | | | | | | | | | | | | | | | | | | Total no. of cells counted |
|---|---|---|---|---|---|---|---|---|---|---|---|---|---|---|---|---|---|---|---|---|---|---|
| | 67 | 68 | 69 | 70 | 71 | 72 | 73 | 74 | 75 | 76 | 77 | 78 | 79 | 80 | 81 | 82 | 83 | 84 | 85 | 86 | 117 | |
| 12 | — | — | — | 1 | — | 3 | 5 | 1 | 3 | 2 | 3 | 2 | 3 | 6 | 3 | 2 | — | — | — | 1 | 1 | 36 |
| 35 | 1 | — | 1 | 1 | 6 | 4 | — | 2 | 2 | — | 1 | — | — | — | — | — | — | — | — | — | — | 18 |

FIG. 6.—Trypsin-Giemsa banded karyotype of a cell with 70 chromosomes at passage 35. 10 marker chromosomes are arranged below the normal chromosomes.

FIG. 7.—Karyotype of a cell with 72 chromosomes at passage 35. 12 marker chromosomes are shown.

from passage numbers 12 and 35 showed some common marker chromosomes, although the average chromosome numbers were reduced in the later passage. The marker chromosomes found were not typical for those found in HeLa cells as reported by Nelson-Rees, Flandermeyer and Hawthorne (1974).

*HL-A typing*

HL-A 2, 3, 7 and 12 were detected on the cultured cells.

*Cryopreservation*

Cells from the first passage onward were recovered successfully with ≥90% viability after frozen storage.

*Mycoplasma contamination*

The cell line was monitored for mycoplasma contamination by a fluorescence method (O'Toole *et al.*, 1976) and by transmission electron microscopy. No contamination was detected.

*Cytotoxicity reactions on cell lines from TCC*

Table II shows a representative experiment in which 4 cell lines from bladder neoplasms and 1 line from non-malignant bladder epithelium were compared in their susceptibility to lysis by lymphoid cells from patients with early stage TCC. Lymphoid cells from the patients with stage T1 or T2 TCC caused significant release of isotope from the lines T24 and J82 from TCC. However, TCCSUP, also of TCC origin, was not affected. Cells from a squamous carcinoma of bladder, SCaBER, and non-malignant urothelium HCV-29 were also not lysed. Lymphoid cells from a normal donor and a patient with urethritis produced no significant lysis of any target.

DISCUSSION

The cell line described derived from an extremely anaplastic transitional cell carcinoma. The histology of the original tumour showed a total replacement of the bladder mucosa and muscle by ulcerated and necrotic tumour. At the time the specimen from the bladder was obtained for tissue culture, the patient already had metastases to the skeleton. Cells from this tumour adapted to *in vitro* culture within a few days. It has been our experience that cells from bladder tumours which metastasize *in vivo* are easily established in long-term tissue culture. Conversely, cells from superficial localized TCC were much slower to grow *in vitro*, and they rarely survived

TABLE II.—*Cytotoxic Effect of Lymphocytes from Bladder Cancer Patients on TCC Target Cells, Assayed by Percentage $^{51}Cr$ Release**

| | Donor | E : T | T24 | J82 | TCCSUP | SCaBER | HCV-29 |
|---|---|---|---|---|---|---|---|
| 1 | TCC | 50 : 1 | 29 | 19 | 6 | 2 | 2 |
| | Stage T1 | 25 : 1 | 12 | 7 | 2 | 4 | 3 |
| 2 | TCC | 50 : 1 | 16 | 12 | 1 | 2 | 5 |
| | Stage T2 | 25 : 1 | 11 | 6 | 0 | 0 | 2 |
| 3 | Urethritis | 50 : 1 | 5 | 1 | 2 | 2 | 0 |
| | | 25 : 1 | 1 | 3 | 1 | 3 | 0 |
| 4 | Normal | 50 : 1 | 2 | 0 | 0 | 3 | 0 |
| | Healthy | 25 : 1 | 1 | 0 | 0 | 0 | 0 |
| * Corrected for spontaneous isotope release: *viz.* | | | 25 | 32 | 31 | 33 | 28 |

E : T = Effector : target cell ratio.
Incubation time 24 h.
T24, J82, TCCSUP cells from TCC. J82 tested in passage 8; TCCSUP in passage 15.
SCaBER cell line from squamous carcinoma bladder, passage 13.
HCV-29 cell line from non-malignant bladder epithelium.

prolonged culture. We have attempted to culture material from urothelial tumours from various anatomical locations, showing varied levels of differentiation. Only 2 of 18 early-stage tumours grew *in vitro*. However, of 3 tumours which rapidly metastasized in the patient, all were successfully established in culture. One of these lines is reported in detail elsewhere (O'Toole *et al.*, 1976). These general observations agree with those of Fogh and Trempe (1975), who described 22 cell lines from human solid tumours which, with a single exception, originated from highly malignant tissue.

The anaplastic nature of the neoplasm from which TCCSUP derived is reflected in the lack of differentiation seen in the cultured cells. The morphology of these cells varied from epithelial to fibroid. The line showed, however, distinct marker chromosomes and a tendency toward hypotetraploidy.

It has previously been observed that lymphoid cells from patients with low-stage TCC selectively destroy cells from TCC (O'Toole *et al.*, 1974). In those experiments, TCC target cells were from the lines T24, RT4 and J82. T24 and J82 were derived from invasive TCC which had not metastasized. RT4 originated from a well differentiated papillary TCC (Rigby and Franks, 1970). These results suggested that these lines expressed common " antigens " which sensitized lymphoid cells from TCC patients recognized. We are developing new cell lines of urothelial origin to investigate the prevalence of shared " antigens " in urothelial tumours. The data presented in Table II show that antigenic differences, as defined by this assay for lymphoid cell cytotoxicity, exist between TCCSUP and 2 other lines from TCC. TCCSUP and a line from a squamous carcinoma of bladder, SCaBER (O'Toole *et al.*, 1976) were not susceptible to lysis by lymphoid cells which lysed T24 and J82. These differences could be quantitative, in that TCCSUP may have a lower density of surface " antigen " and hence be not readily recognized by sensitized lymphoid cells. Alternatively, TCCSUP may not express the relevant components which are present in T24, J82 and RT4 cells. The biological relevance of such differences between malignant cells derived from a common histological origin remains to be determined.

We thank Dr. J. Waisman, Department of Pathology, UCLA School of Medicine for providing the histology on the original tumour specimen. This study was supported by Grant 16880 from the National Bladder Cancer Project.

REFERENCES

BATTIFORA, H., EISENSTEIN, R. & McDONALD, J. H. (1964) The Human Urinary Bladder Mucosa. An Electron Microscopic Study. *Invest. Urol.*, **1**, 354.

BUBENIK, J., BARESOVA, M., VIKLICKY, C., JAKOUBKOVA, J., SAINEROVA, H. & DONNER, J. (1973) Established Cell Line of Urinary Bladder Carcinoma (T24) Containing Tumor-specific Antigen. *Int. J. Cancer*, **11**, 765.

BURROWS, M. T., BURNS, J. E. & SUZUKI, Y. (1917) Cultivation of Bladder and Prostatic Tumors Outside the Body. *Johns Hopkins Hosp. Bull.*, **28**, 178.

ELLIOT, A. Y., CLEVELAND, P., CERVENKA, J., CASTRO, A. E., STEIN, N., HAKALA, T. R. & FRALEY, E. E. (1974) Characterization of a Cell Line from Human Transitional Cell Carcinoma of the Urinary Tract. *J. natn. Cancer Inst.*, **53**, 1241.

FOGH, J. & TREMPE, G. (1975) New Human Tumor Cell Lines. In *Human Tumor Cells In Vitro*. Ed. J. Fogh. New York: Plenum Press. p. 115.

GEY, G. O., COFFMAN, W. D. & KUBIECK, M. J. (1952) Tissue Culture Studies of the Proliferative Capacity of Cervical Carcinoma and Normal Epithelium. *Cancer Res.*, **12**, 266.

HAKALA, T. R. & LANGE, P. (1974) Serum Induced Lymphoid Cell Mediated Cytotoxicity against Human Transitional Cell Carcinomas of the Genito-urinary Tract. *Science, N.Y.*, **184**, 795.

NELSON-REES, W. A., FLANDERMEYER, R. R. & HAWTHORNE, P. K. (1974) Banded Marker Chromosomes as Indicators of Intraspecies Cellular Contamination. *Science, N.Y.*, **184**, 1093.

O'TOOLE, C., STEJSKAL, V., PERLMANN, P. & KARLSSON, M. (1974) Lymphoid Cells Mediating Tumor-specific Cytotoxicity to Carcinoma of the Urinary Bladder. Separation of the Effector Population Using a Surface Marker. *J. exp. Med.*, **139**, 457.

O'TOOLE, C., NAYAK, S., PRICE, Z., GILBERT, W. H. & WAISMAN, J. (1976) A Cell Line (SCaBER) Derived from Squamous Cell Carcinoma of the

Human Urinary Bladder. *Int. J. Cancer*, **17**, 707.

PRICE, Z. H. (1972) A Three Dimensional Model of Membrane Ruffling from TEM and SEM of Cultured Monkey Kidney Cells ($LLCMK_2$). *J. Microscopy*, **95**, 493.

RIGBY, C. C. & FRANKS, L. M. (1970) A Human Tissue Culture Cell Line from a Transitional Cell Tumor of the Urinary Bladder: Growth, Chromosome Pattern and Ultrastructure. *Br. J. Cancer*, **24**, 746.

RUSSELL, W. C., NEWMAN, C. & WILLIAMSON, D. H. (1975) A Simple Cytochemical Technique for Demonstration of DNA in Cells Infected with Mycoplasms and Virus. *Nature, Lond.*, **253**, 461.

SEABRIGHT, M. (1971) A Rapid Banding Technique for Human Chromosomes. *Lancet*, ii, 971.

SPRINGER, E. L., HACKETT, A. J. & NELSON-REES, W. A. (1976) Alteration of the Cell Membrane Architecture during Suspension and Monolayer Culturing. *Int. J. Cancer*, **17**, 407.